# Triglyceride glucose-body mass index is effective in identifying nonalcoholic fatty liver disease in nonobese subjects

**DOI:** 10.1097/MD.0000000000007041

**Published:** 2017-06-02

**Authors:** Shujun Zhang, Tingting Du, Mengni Li, Jing Jia, Huiming Lu, Xuan Lin, Xuefeng Yu

**Affiliations:** a Division of Endocrinology, Department of Internal Medicine, Tongji Hospital, Tongji Medical College, Huazhong University of Science and Technology; b Department of Health Examination; c Department of Endocrinology, Wuhan Iron and Steel Company (WISCO) General Hospital, Wuhan, Hubei Province, China.

**Keywords:** body mass index, fasting plasma glucose, insulin resistance, triglyceride

## Abstract

Nonalcoholic fatty liver disease (NAFLD) is an increasingly common condition that is highly correlated with obesity; however, it is not uncommon among nonobese individuals. Triglyceride (TG) and glucose index combined with body mass index (TyG-BMI) has been proposed as a favorable marker of insulin resistance. We sought to investigate the effectiveness of TyG-BMI in identifying NAFLD in nonobese subjects.

We conducted a cross-sectional study in a nonobese (BMI <25.0 kg/m^2^) Chinese population (N = 6809) of adults who underwent health examinations, including abdominal ultrasonography.

The prevalence of ultrasonography-detected NAFLD was 23.9% in nonobese subjects. After adjusting for potential confounders, every 1-standard deviation increase in TyG-BMI had an odds ratio (OR) of 3.4 [95% confidence interval (95% CI), 3.0–3.9] for NAFLD. Compared with the lowest quartile of TyG-BMI, multivariable-adjusted ORs were 2.4 (1.6–3.6), 6.4 (4.2–9.7), and 15.3 (9.8–23.9) for those in the second, third, and fourth quartile, respectively. According to the receiver operating characteristic curve analysis, TyG-BMI was effective in diagnosing patients with NAFLD with an area under the curve of 0.835 (95% CI, 0.824–0.845). In comparison, TyG-BMI was superior to its components, including TyG, BMI, TG, and fasting plasma glucose, for identifying nonobese subjects at risk for NAFLD.

In this study, the prevalence of NAFLD was over one-fifth in the nonobese population. TyG-BMI was an effective marker to detect NAFLD in nonobese subjects.

## 1 Introduction

Nonalcoholic fatty liver disease (NAFLD) is an increasingly common condition worldwide, with a prevalence of 15% to 40% in the general population.^[1–3]^ The rise of NAFLD has become a public health concern. It is associated with an increased risk for cardiovascular disease and mortality from liver-related and liver-unrelated causes.^[4–6]^ NAFLD is frequently seen in individuals with metabolic abnormalities associated with obesity. However, not all obese individuals develop NAFLD.^[7]^ In fact, NAFLD can be found in nonobese individuals.^[8]^ This condition refers to lean or nonobese NAFLD.^[9]^

Although NAFLD is more prevalent in obese individuals, nonobese patients with NAFLD are not uncommon. Epidemiological data indicate that 10% to 30% of nonobese individuals have evidence of hepatic steatosis, and therefore, nonobese NAFLD.^[3,9,10]^ Notably, nonobese NAFLD seems to more commonly prevail among Asians than among other populations. The prevalence and severity of nonalcoholic steatohepatitis and fibrosis in nonobese individuals with NAFLD was not significantly different from that in obesity-related NAFLD.^[11,12]^ Moreover, nonobese individuals with NAFLD may represent a subset of NAFLD in metabolically obese but normal-weight individuals with metabolic abnormalities similar to obesity-related metabolic profiles.^[13]^ These patients exhibit a high incidence of diabetes, cardiovascular disease, and all-cause mortality.^[14,15]^ Therefore, it may be important to identify high-risk nonobese patients and manage their metabolic profile. However, data regarding the risk factors and impact of nonobese NAFLD remain incomplete; especially, few studies were conducted on exploring biomarkers in identifying nonobese NAFLD patients.

The product of triglycerides and glucose, TyG, was suggested as a favorable surrogate marker for insulin resistance.^[16,17]^ Previous studies also reported that the TyG index was useful in identifying various metabolic abnormalities associated with insulin resistance, such as type 2 diabetes,^[18]^ the metabolically obese but normal weight phenotype,^[19]^ and NAFLD.^[20]^ In addition, a recent study revealed that TyG combined with body mass index (BMI) was a more efficient marker for insulin resistance than other indicators.^[21]^ Insulin resistance is as important in nonobese individuals with NAFLD as it is in individuals with obesity-related NAFLD. Previous studies have also suggested that insulin resistance may have an even stronger association with NAFLD in nonobese individuals than in obese ones.^[22]^ To the best of our knowledge, limited data are available regarding the effectiveness of TyG-BMI in recognizing individuals at risk for nonobese NAFLD.

Therefore, in this study, we sought to examine the association between TyG-BMI and NAFLD risk in nonobese Chinese individuals and evaluate the performance of TyG-BMI in identifying NAFLD in the population.

## 2 Methods

### 2.1 Subjects and study design

The study participants were Chinese employees (age >20 years) from the Wuhan Iron and Steel Company (WISCO), which is one of the largest iron and steel companies in China. The data were obtained from health examinations of all employees and retired workers undertaken at the WISCO General Hospital in 2009. Questionnaires including demographic characteristics, such as age, sex, medical history, family history, and drinking status were collected. We used the data of nonobese subjects with BMI <25.0 kg/m^2^, according to the Wealth Health Organization criteria for Asians.^[23]^ The exclusion criteria included hepatic virus infection carriers, autoimmune hepatic disease, other chronic hepatic diseases; taking diabetes or dyslipidemia medication or antihypertensive medication; missing data on age, sex, blood pressure (BP), BMI; and fasting plasma glucose (FPG), triglyceride (TG), alanine aminotransferase (ALT), or ultrasonography examination of the liver. Altogether, 6809 participants were included in the analysis, including 4058 men and 2751 women. This study was approved by the institutional review board of WISCO General Hospital and it conforms to the provisions of the Declaration of Helsinki. We were exempt from the informed consent requirement because of retrospective estimation of de-identified database.

### 2.2 Clinical measurements

As our previous studies described,^[24–26]^ physical examination was performed and anthropometry was obtained, including weight, height, and BP. BMI was calculated as weight (kg)/square of height (m^2^). BP was measured following standardized protocols from the World Health Organization (WHO) by trained examiners using a mercury sphygmomanometer with appropriate cuff at 3 different consecutive times at 3 to 5-min intervals on 1 visit. The 3 readings were averaged as the BP values in our data analysis. Blood samples were collected after a fast of at least 10 hours overnight and analyzed for biochemical measurements, such as ALT, FPG, uric acid, white blood cell count, hepatitis viral antigen/antibody, and serum lipids, including TG, total cholesterol, high-density lipoprotein cholesterol, and low-density lipoprotein cholesterol. All the measurements were determined by an auto-analyser (Hitachi 7600, Ltd, Tokyo, Japan). The TyG index was calculated as established formulas, TyG = Ln [TG (mg/dL) × FPG (mg/dL)/2].^[17,27]^ TyG-BMI indicates TyG × BMI.^[21]^

### 2.3 Definition for NAFLD

According to the guidelines proposed by the Asia-Pacific Working Party,^[28]^ patients were diagnosed with NAFLD if they had a fatty liver, and did not exhibit excessive alcohol intake (>140 g/week for men, >70 g/week for women), did not have a history of carrying hepatic virus, and did not use steatogenic or hepatotoxic medications. Fatty liver was assessed as the presence or absence of hepatic steatosis by ultrasound scan, identified by 1 professional operator using a standard method (i.e., the presence of increased echogenicity of liver, compared with renal cortex).

### 2.4 Statistical analysis

All statistical analyses were performed using SPSS version 20.0 (SPSS Inc., Chicago, IL). Normality testing was conducted, and continuous variables expressed as median and interquartile ranges (IQRs) because of their skew distribution, whereas categorical variables were presented as percentages. Differences between NAFLD and non-NAFLD individuals were assessed using the Mann–Whitney *U* test for continuous variables and Chi-square test for categorical variables.

Binary logistic regression analysis was conducted to calculate odds ratios (ORs) and 95% confidence intervals (95% CIs) for NAFLD in quartiles of TyG, BMI, or TyG-BMI. The multivariable-adjusted ORs and corresponding 95% CIs for NAFLD associated with 1-standard deviation (SD) increase of TyG-BMI and its components were further estimated in the population. The adjusted variables were derived from significant results of univariable analysis with *P* < .1. Finally, we performed the receiver operating characteristic (ROC) curve analysis to test the ability of TyG-BMI to diagnose patients with NAFLD. Comparisons between the areas under the ROC curve (AUC) of TyG-BMI and its components were conducted using the method described by DeLong et al.^[29]^ A 2-tailed *P* value <.05 was considered significant.

## 3 Results

### 3.1 Characteristics of the study population

The study population had a mean (±SD) age of 48.4 ± 15.1 years and a mean BMI of 22.0 ± 2.0 kg/m^2^. The prevalence of ultrasonography-diagnosed NAFLD was 23.9% in the nonobese population. Characteristics of subjects diagnosed with NAFLD are described in Table 1. Compared with non-NAFLD individuals, patients with NAFLD were more likely to be older, men, and have a worse metabolic profile, including BMI, BP, FPG, uric acid, serum lipids, and TG/HDL ratio (all *P* < .0001). Notably, the levels of TyG index and TyG-BMI were significantly higher in patients with NAFLD, than in subjects without the disease (both *P* < .0001).

**Table 1:**
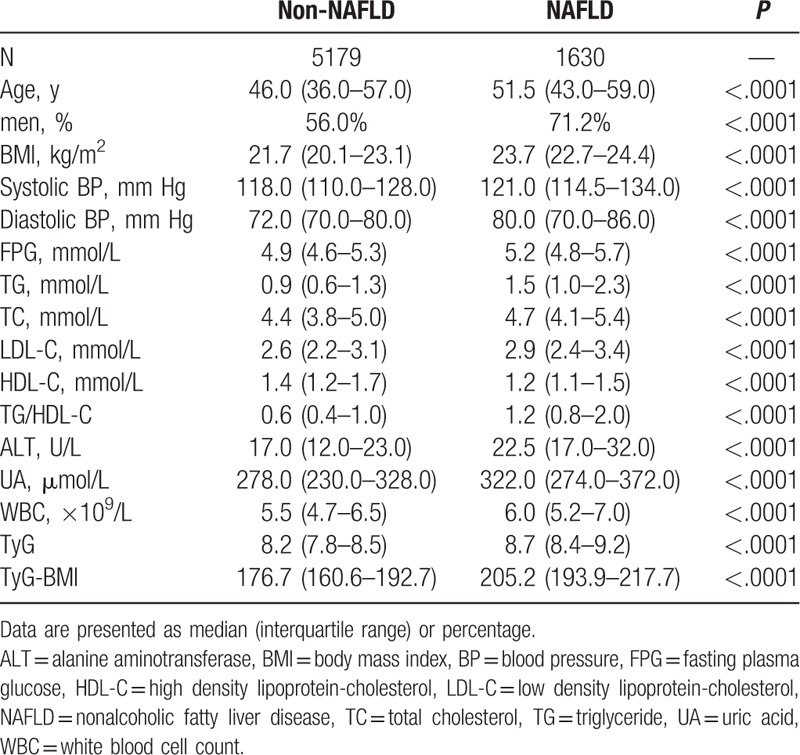
Characteristics of the population according to NAFLD status.

### 3.2 The association between TyG-BMI and NAFLD risk

Subjects were divided into 16 groups on the basis of TyG or TG and BMI quartiles. We found that TyG combined with BMI identified individuals at a higher risk for NAFLD (Table 2). For a given quartile of TyG, the multivariable-adjusted ORs for NAFLD risk gradually increased with increasing BMI quartiles. Similarly, ORs progressively increased across TyG quartiles in each BMI quartile. Consequently, the highest TyG quartile combining with the highest ALT quartile showed the strongest association with risk for NAFLD (*P* for interaction = .016). The similar results were seen for TG combining with BMI (*P* for interaction = .023).

**Table 2:**
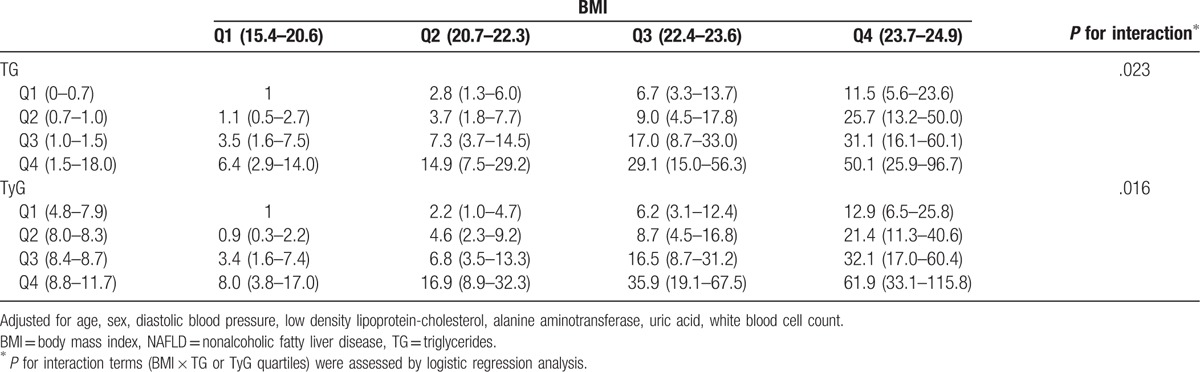
Odds ratios for NAFLD according to categories of BMI and TG or TyG quartiles.

When categorizing TyG-BMI into quartiles, we observed a dose–response association between TyG-BMI and NAFLD. The prevalence of NAFLD increased from 2.2% to 9.0% to 27.8% to 56.7% across the increasing TyG-BMI quartiles (*P* for trend <.0001). ORs for NAFLD risk increased with increasing TyG-BMI in different models (Table 3).

**Table 3:**
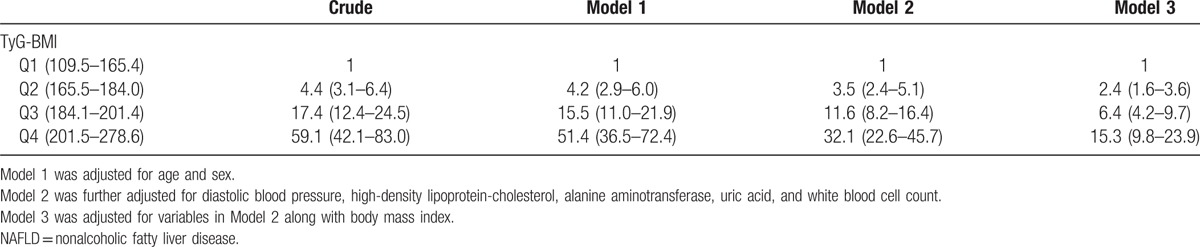
Odds ratios for NAFLD in quartiles of TyG-BMI.

The crude ORs and 95% CIs for NAFLD were 4.4 (3.1–6.4), 17.4 (12.4–24.5), and 59.1 (42.1–83.0) for subjects in the second, third, and fourth quartile of TyG-BMI, respectively, compared with those in the first quartile. ORs were decreased; however, the results remained significant after adjusting for age and sex (model 1). The associations persisted, albeit attenuated, after adjusting for diastolic BP, high-density lipoprotein cholesterol, ALT, uric acid, white blood cell count (model 2), and BMI (model 3).

Table 4 summarizes the ORs for NAFLD for every 1-SD increment of TyG-BMI or its components in the population. In the crude model, every 1-SD increase of TyG-BMI had an OR of 4.9 (95% CI, 4.4–5.3) for NAFLD. Adjusting for all covariates resulted in an OR of 3.4 (95% CI, 3.0–3.9). The ORs for NAFLD of every 1-SD increase in TyG, BMI, TG, and FPG were also significant, but relatively weaker than that of TyG-BMI.

**Table 4:**
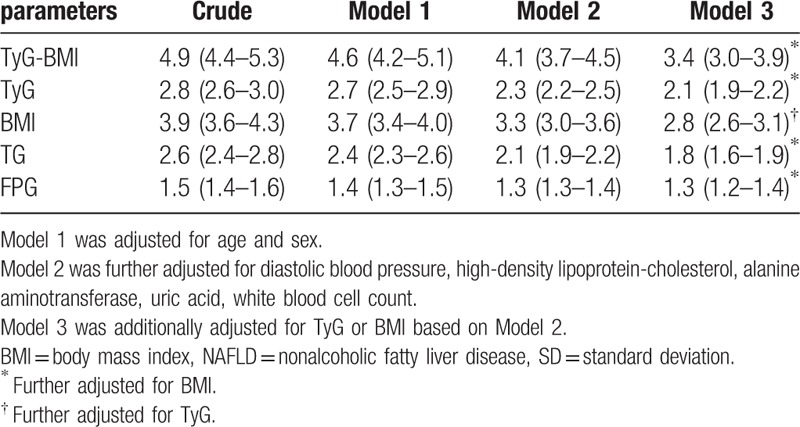
Odds ratios for NAFLD of 1-SD increase in TyG-BMI and its components.

### 3.3 Performance of TyG-BMI and its components for diagnosing patients with NAFLD

We further conducted a ROC curve analysis to assess the diagnostic value of TyG-BMI for NAFLD and simultaneous comparison with its components. The AUC derived from TyG-BMI and its components are presented in Table 5. The AUC of TyG-BMI for discriminating NAFLD was 0.835 (95% CI 0.824–0.845), which was greater than that of all components. The AUC of BMI [0.783 (95% CI 0.771–0.794)] was greater than that of TyG, TG, and FPG.

**Table 5:**
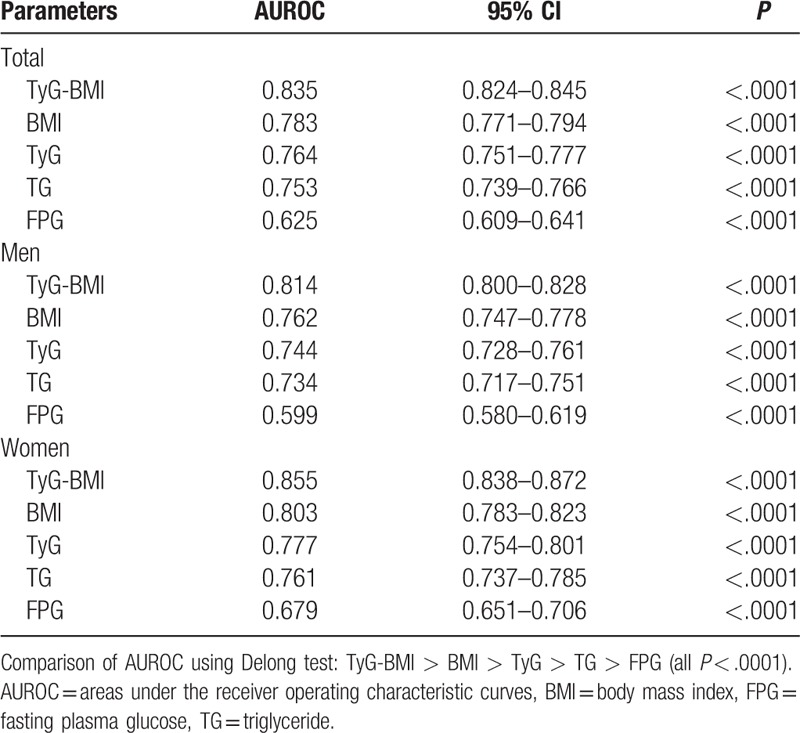
Areas under the receiver operating characteristic curves (AUROC) for each evaluated parameters in identifying nonalcoholic fatty liver disease.

In subgroup analysis, TyG-BMI was still superior to other indicators in diagnosing NAFLD in both men and women.

## 4 Discussion

In this study, we observed that NAFLD was prevalent in more than one-fifth of the nonobese Chinese population. Hence, the identification of an effective marker to screen for NAFLD in nonobese individuals is of great significance. Our findings indicated that there was a strong and positive association between TyG-BMI and risk for NAFLD, after adjusting for potential confounders. We also demonstrated that TyG-BMI could identify NAFLD accurately with an AUC of 0.835 (95% CI, 0.824–0.845), which was greater than that of its components, including TyG, BMI, TG, and FPG. On the basis of these results, we suggest that TyG-BMI is an effective marker for identifying nonobese patients with NAFLD.

We observed a relatively high prevalence of nonobese patients with NAFLD in this population, which was comparable to other studies. The prevalence of ultrasonography-detected hepatic steatosis in nonobese (BMI <30 kg/m^2^) participants reported from the US National Health and Nutrition Examination Survey (NHANES) was 27%.^[9]^ NAFLD was present in 27% of nonobese (BMI <25 kg/m^2^) and nondiabetic subjects in Korea.^[10]^ A population-based study in Hong Kong reported the prevalence of nuclear magnetic resonance spectroscopy detected hepatic steatosis was 19.3% in nonobese (BMI <25 kg/m^2^) subjects.^[30]^ On the basis of the differences in study subject selection, diagnostic modalities, nutritional status of the specific population, and recommended BMI threshold for the East and West, the global reported prevalence of nonobese NAFLD varies widely. The nonobese population in this study was from WISCO, which is one of the largest iron and steel companies in China; hence, the results in our study may reflect a general level of NAFLD prevalence among nonobese Chinese workers. We found that more than one-fifth of nonobese individuals may have NAFLD; therefore, more attention must be paid to nonobese Chinese individuals, especially Chinese employees. It is important to find a simple and cost-effective marker to screen for NAFLD in nonobese individuals.

In this study, TyG-BMI incorporating TG, FPG, and BMI showed an excellent performance in identifying ultrasonography-detected NAFLD in nonobese individuals. Recent studies have demonstrated that the TyG index, a surrogate marker for insulin resistance, was effective in identifying individuals at risk for NAFLD.^[20,31]^ As it was derived from TG and FPG, these are 2 crucial metabolic variables that are altered in the fatty liver and highly correlated with insulin resistance.^[16,17]^ It is believed that insulin resistance and its related metabolic risk factors still play an important role in the development of NAFLD in nonobese subjects as noted in the development of obese-associated NAFLD. Kwon et al^[22]^ suggested that nonobese NAFLD even had a stronger association with metabolic disorders than obese NAFLD. Therefore, the TyG index may have a superior performance in recognizing NAFLD in nonobese participants than in obese ones. Our data of the whole population including nonobese and obese subgroups showed that TyG was indeed more closely associated with NAFLD risk in nonobese individuals than in obese ones (data not shown).

Interestingly, our results further demonstrated that TyG-BMI was superior to TyG in identifying NAFLD in nonobese patients. These results indicate that BMI plays a vital role in the development of NAFLD in nonobese people. It is likely because being overweight (23 kg/m^2^ ≤BMI <25 kg/m^2^) is also closely correlated with NAFLD risk.^[10]^ However, when the data were reanalyzed, we observed that the prevalence of NAFLD was 11.7% in subjects with BMI <23 kg/m^2^. This suggests that even a subtle increase of BMI within the normal range could be a risk factor for NAFLD. Our observation in Table 2 that the risk for nonobese NAFLD increased with the elevation of BMI levels regardless of TyG quartiles upholds the strong relationship between BMI and nonobese NAFLD. Moreover, it is increasingly recognized that the distribution of fat in the abdominal area, especially visceral abdominal adiposity, plays a much more important role in the development of nonobese NAFLD. Recent studies reported that nonobese NAFLD subjects have higher body fat content and evidence of visceral adiposity than non-NAFLD individuals with a comparable BMI in Asia.^[8]^ Furthermore, Chinese individuals have a greater amount of visceral adipose tissue than Europeans, even with the same BMI.^[32]^ This may also explain why a normal BMI was still an independent risk factor for NAFLD in our study; these results are concurrent with those of previous studies.^[8,30]^ Therefore, BMI is useful in identifying NAFLD in nonobese individuals, and it is reasonable to conclude that TyG-BMI is more efficient than the TyG index alone for recognizing nonobese patients with NAFLD.

Our study has the following limitations. First, we diagnosed NAFLD using ultrasonography, which has limited sensitivity and does not reliably detect liver fat infiltration <20%.^[33]^ Liver biopsy remains the gold standard for diagnosing patients with NAFLD; however, it was impractical in this case, because it is invasive. Noninvasive tools have been developed to detect NAFLD, including ultrasonography, computed tomography, and magnetic resonance spectroscopy. The latter 2 tools are expensive and time-consuming; thus, ultrasound is recommended as the first-line imaging technique to screen patients for NAFLD in the clinical setting. Second, the study was cross-sectional in design; therefore, a causal relationship cannot be obtained. The participants comprised selected populations (industrial employees and retired workers) with a preponderance of men. Therefore, extrapolating these findings to the Chinese general population or other races or ethnicities should be interpreted cautiously. Third, information about body fat distribution was unavailable in this study; hence, we could not compare the efficacy between TyG-BMI and other clinical indexes, such as fatty liver index. Finally, the ability of TyG-BMI in detecting nonalcoholic steatohepatitis and advanced fibrosis could not be evaluated; this must be assessed by future studies.

In conclusion, our study showed that TyG-BMI was strongly associated with increased risk for Chinese subjects with nonobese NAFLD. TyG-BMI performed better than its components to identify nonobese patients with NAFLD. This finding is of particular clinical relevance, as TyG-BMI can be calculated from common measures, which are widely available and based on standardized measurements.

## Acknowledgment

We thank all the participants for their contribution and participation.
